# Molecular mechanisms underlying Parkinson’s disease and role of phytochemicals, α-synuclein, sirtuins, and incretin mimetics in potential therapy

**DOI:** 10.3389/fphar.2026.1798309

**Published:** 2026-03-26

**Authors:** Sanjida Shahid Juthi, Prawej Ansari, Md Ferdos Ahmed, Joyeeta T. Khan, Veronique Seidel, Sandeep Kumar, Reena Kumari, Aine McKillop, Yasser H. A. Abdel-Wahab, Peter R. Flatt

**Affiliations:** 1 Behavioral Services, Anderson Center for Autism, Staatsburg, NY, United States; 2 Centre for Diabetes Research, School of Biomedical Sciences, Ulster University, Coleraine, United Kingdom; 3 Department of Pharmacology, National Medical College and Teaching Hospital, Birgunj, Nepal; 4 Department of Pharmacy, School of Pharmacy and Public Health, Independent University, Bangladesh (IUB), Dhaka, Bangladesh; 5 Department of Genetics, Comprehensive Diabetes Center, Heersink School of Medicine, University of Alabama, Birmingham, AL, United States; 6 Natural Products Research Laboratory, Strathclyde Institute of Pharmacy and Biomedical Sciences, University of Strathclyde, Glasgow, United Kingdom; 7 Department of Microbiology and Immunology, Tulane University, New Orleans, LA, United States

**Keywords:** GLP-1, incretin mimetics, neurodegeneration, Parkinson’s disease, phytoconstituents, sirtuins, α-synuclein

## Abstract

Parkinson’s disease (PD) is a progressive neurodegenerative disorder primarily characterized by dopaminergic neuronal loss in the substantia nigra and intracellular accumulation of misfolded α-synuclein aggregates. Despite being extensively studied, current pharmacological and surgical interventions remain mostly symptomatic, leaving limited efficacy in halting or reversing disease progression. The complicated pathogenesis of PD involves oxidative stress, mitochondrial dysfunction, neuroinflammation, impaired autophagy, and proteostasis imbalance, altogether contributing to neuronal vulnerability. In addition to established drugs such as levodopa, other medications affecting dopamine pathways and incretin mimetics, numerous studies have highlighted the therapeutic potential of phytomolecules to target these processes. This includes resveratrol, curcumin, quercetin, baicalein, berberine and epigallocatechin gallate (EGCG), which have demonstrated pleiotropic neuroprotective effects by mitigating oxidative and inflammatory cascades, improving mitochondrial biogenesis, preventing proteostasis imbalance, and/or blocking α-synuclein aggregation. Some phytomolecules may also act through the sirtuin and PI3K/Akt signaling pathways, linking neuroprotection with metabolic regulation. Some phytomolecules may additionally alleviate insulin resistance and stimulate incretin (GLP-1/GIP) secretion, potentially enhancing their neuroprotective. The exact relationship between αS, sirtuins, insulin signaling and phytomolecules is not yet fully understood. Nevertheless, increasing evidence suggests that phytomolecules can modulate brain insulin resistance and enhance incretin signaling, which contribute to their neuroprotective effects in PD. This review highlights the interconnected metabolic and neuronal mechanisms in Parkinson’s disease encompassing α-synuclein pathology, sirtuin imbalance, and disrupted insulin signaling role in PD and explores incretin and phytomolecules molecule based therapies, often utilized for type 2 diabetes management as complementary multi-target neuroprotective strategies.

## 1 Introduction

Parkinson’s disease (PD) is a neurodegenerative condition associated with progressive neuronal loss in the substantia nigra pars compacta (SNpc), the major origin of dopamine release in the brain (Ramesh and Arachchige, 2023). This deficiency in dopamine triggers tremors, rigidity, slowness of movement, and problems with balance and coordination (Zafar and Yaddanapudi, 2025). PD affects roughly 0.3% of the general population, with prevalence rising to about 1% among individuals over 60 years of age (Dorsey et al., 2018). Between 1990 and 2015, the worldwide prevalence of PD almost doubled, and with the increasing growing aging population, it has been estimated that this figure will reach 12 million by 2040 (Li et al., 2025).

The current therapeutic options for the management of PD aim to increase dopamine concentration at the synaptic level for symptomatic relief of motor deficits. The first-line drug for the management of PD is the dopamine replacement agent levodopa. As PD progresses, patients often need higher or more frequent dosing of this medication, which is limited by adverse effects such as dyskinesias (Vijiaratnam et al., 2021; Pardo-Moreno et al., 2023; Oertel and Schulz, 2016). Levodopa is often formulated in combination with decarboxylase inhibitors (e.g., carbidopa, benserazide) to enhance its central bioavailability. However, the use of combined therapies also increases adverse side effects (Müller, 2015). Other drugs, such as monoamine oxidase B (MAO-B) inhibitors (e.g., rasagiline, safinamide, selegiline) or catechol-O-methyltransferase (COMT) inhibitors (e.g., entacapone, tolcapone), may also be used to improve dopamine levels, although sometimes symptoms may persist or worsen even with optimized dopaminergic therapy (Pardo-Moreno et al., 2023; Oertel and Schulz, 2016; Müller, 2015; Dhanawat et al., 2020; Smith et al., 2021; Alfaidi et al., 2024).

The protein α-synuclein (αS) is the key biological marker for PD. The accumulation of misfolded or oligomeric αS aggregates in the brain contributes to the observed neurodegeneration. The accumulation of αS can downregulate the expression of other proteins called sirtuins (Sirts) (Srinivasan et al., 2021). Interestingly, studies have highlighted a link between PD and impaired insulin signaling pathways which can reduce sirtuin activity, exacerbate mitochondrial dysfunction, and promote αS aggregation. Understanding this metabolic-neuronal axis and the mechanisms underlying αS accumulation and sirtuins dysregulation may provide novel insights into potential therapeutic targets for PD (Alfaidi et al., 2024; Srinivasan et al., 2021; Giguère et al., 2018).

Furthermore, mitochondrial dysfunction, neuroinflammation, and oxidative stress are central to biochemical events in PD such as αS aggregation, dysregulated sirtuin activity, and impaired insulin signaling (Picca et al., 2020). These interconnected mechanisms can trigger dopaminergic neuronal degeneration and contribute to the progressive nature of PD (Ruiz-Pozo et al., 2023). Special emphasis in emerging research has been placed on phytomolecules and incretin (Glucagon like peptide-1 or, GLP-1) mimetics, which have demonstrated neuroprotective and metabolic regulatory effects in preclinical PD models (Lv et al., 2024). Plant-derived compounds such as resveratrol (*Polygonum cuspidatum*), epigallocatechin gallate (EGCG; *Camellia sinensis*), quercetin (*Allium cepa, Malus domestica*), baicalin (*Scutellaria baicalensis*), and berberine (*Berberis spp*.) have been reported to suppress αS aggregation, improve mitochondrial function, and preserve dopaminergic neurons by modulating sirtuin and PI3K/Akt signaling pathways (Roy et al., 2024; Valenti et al., 2016; Brimson et al., 2021).

This article delineates the mechanistic interplay in PD between αS pathology, sirtuin dysregulation, and impaired insulin signaling, illustrating how systemic metabolic dysfunction may contribute to the progression of neurodegeneration. Within this context, it further considers incretin mimetics and phytomolecules, therapeutic strategies established in Type 2 Diabetes management as complementary, multi-target pharmacological approaches with potential to stabilize neuronal energy metabolism, restoring mitochondrial homeostasis, and mitigate αS-associated neurodegenerative processes.

## 2 Methodology

This review was conducted through a literature search in PubMed, Scopus, Web of Science and Google Scholar, using combinations of keywords such as *“Parkinson’s disease,” “α-synuclein aggregation,” “sirtuins,” “insulin signaling,” GLP-1* and *incretin mimetics “phytomolecules,” “neuroprotection,”* and *“therapeutics.”* The search covered publications from 2010 to January 2026. Initially, about 640 articles were identified; after removal of duplicates and screening of titles and abstracts, 320 articles were evaluated in full text. Following the application of inclusion criteria (peer-reviewed studies, systematic reviews, meta-analyses and preclinical or clinical studies in English) and excluding conference abstracts, non-peer-reviewed material and studies lacking mechanistic or therapeutic relevance, a final set of 124 studies was included. The studies included in this review focused on key molecular mechanisms involved in PD, such as α-synuclein aggregation, sirtuin dysregulation and impaired insulin signaling. In addition, neuroprotective strategies targeting mitochondrial dysfunction, oxidative stress, and neuroinflammation and the effects of phytomolecules polyphenols, flavonoids, alkaloids, terpenoids -and GLP-1 mimetics, which have demonstrated significant neuroprotective and metabolic regulatory efficacy in preclinical and clinical models, are included. This structured approach enabled a broad but integrated synthesis of evidence, combining therapeutic strategies and molecular mechanistic insights. As a result, it is systematically highlighting the interrelationships between metabolic regulation, neurodegeneration, phytochemical-based therapies in PD, and provides a solid foundation for understanding current research and emerging approach. This approach ensured a comprehensive yet focused synthesis of evidence on α-synuclein, sirtuins, insulin signaling, GLP-1 and incretin mimetics and the therapeutic potential of phytomolecules in PD.

## 3 The role of αS in PD

Native αS is a small monomeric presynaptic protein of 140 amino acids involved in vesicle trafficking, docking and fusion as well as neurotransmitter release and regulation, microtubule formation and axonal transport (Giguère et al., 2018; Espay et al., 2019; Singleton et al., 2003; Tan et al., 2000; Kalia, 2019). Besides its role in the synaptic machinery, αS is also important for ATP production, mitochondrial trafficking and morphology (Vicario et al., 2018), and the regulation of mitophagy-related genes such as PRKN, PINK1, and DJ-1 (Braak et al., 2003; Wong et al., 2019; Ludtmann et al., 2018). PD is defined as an α-synucleinopathy, whereby misfolded or oligomeric αS aggregates accumulate in the mitochondrial membranes. This disrupts mitochondrial membrane potential (MMP), leading to mitochondrial dysfunction, mitophagy dysregulation, neuroinflammation, and subsequent dopaminergic neuronal degeneration (Meng et al., 2025). The presence of increased levels of oxidized dopamine and lower glucocerebrosidase activity, in turn promotes αS accumulation (Burbulla et al., 2017).

Although direct interconnections between the release of pro-inflammatory cytokines and αS accumulation have yet to be established, altered pro-inflammatory cells in experimental PD models suggest that inflammation and αS dysregulation are closely interconnected (Tan et al., 2020). Several studies have indicated that the presence of αS aggregates in the gut can spread out to the brain via the vagus nerve (Breen et al., 2019). One study carried out in αS transgenic mice demonstrated that antibiotic treatment could ameliorate motor symptoms, with symptoms subsequently aggravated following microbial recolonization (Kim et al., 2019). These findings suggests that gut microbiota and dysbiosis, infection and inflammation are crucial triggers for αS dysregulation and the pathogenicity of PD.

αS has a high binding affinity for pre-synaptic vesicles and its accumulation in pre-synaptic terminals is a sign of synaptopathy. Ultimately, after being released into the synaptic space, it is taken up by surrounding neurons and glia, resulting in neurodegeneration (Burré et al., 2014; Bridi and Hirth, 2018; Lee et al., 2010; Peng et al., 2018). Beyond its role in neurotransmitter release, αS has also been implicated in modulating neuronal excitability and firing or signaling patterns (Janezic et al., 2013; Subramaniam et al., 2014). Interestingly, research carried out on different neuroanatomical regions has revealed that neuronal and glial cell populations have varying susceptibility to αS toxicity. This has been attributed to differences in neuronal circuitry linked to the complex interplay of genetics, metabolic stress and environmental factors, which helps explain why PD emerges and develops in different ways in different individuals (Wong and Krainc, 2017; Alegre-Abarrategui et al., 2019; Brezic et al., 2025). Collectively, these findings demonstrate that αS dysregulation is a primary pathogenic driver in PD, triggering synaptic dysfunction, mitochondrial impairment followed by neuroinflammation via ROS, ultimately leading to dopaminergic neuronal loss. As such, αS remains a compelling and highly prioritized target for disease-modifying therapeutic intervention.

## 4 The role of Sirts in PD

Sirtuins (Sirts) are proteins that belong to the nicotinamide adenine dinucleotide (NAD^+^)-dependent deacetylases family (Histone Deacetylase or HDAC). There are seven different types of sirtuins (Sirt1-7). These proteins play an important role in DNA damage repair, autophagy, apoptosis, energy metabolism, inflammatory response and mitochondrial health. Sirt1, Sirt6 and Sirt7 are predominantly localised in the nucleus, while Sirt2 is found in the cytoplasm, and Sirt3, Sirt4 and Sirt5 are found in the mitochondria (Yu et al., 2022).

Sirt1 and Sirt2 are crucial for mitochondrial biogenesis (Saihara et al., 2017; Chuang et al., 2019). Sirt2 also regulates neurite outgrowth in sensory neurons (Gupta et al., 2022). Both modulate transcription factors such as FoxO3a, influencing cellular responses to stress, apoptosis and autophagy. Sirt3, Sirt4, and Sirt5 are primarily localized in mitochondria, where they regulate mitochondrial metabolism and biogenesis. Sirt3-mediated deacetylation governs the electron transport chain, ATP synthesis, and antioxidant defense by activating superoxide dismutase (SOD) and maintaining mitochondrial DNA integrity. Similarly, Sirt4 regulates lipid metabolism and metabolic flux through its influence on malonyl CoA decarboxylase and pyruvate dehydrogenase, while also modulating mitochondrial signaling via AMPK-PGC1α and PPARα pathways. Sirt5 contributes to mitochondrial homeostasis by controlling the urea cycle and enzymatic acetylation, thereby influencing energy metabolism, glucose turnover and oxidative balance (Braidy et al., 2019; Laurent et al., 2013; Mathias et al., 2014; Carrico et al., 2018)). Sirt6 and Sirt7 are nuclear enzymes that also contribute to mitochondrial biogenesis and genomic stability. Sirt6 enhances mitochondrial gene expression through AMPK-PGC1α activation, while Sirt7 regulates mitochondrial energy metabolism through post translational modification of nuclear proteins (Song et al., 2022; Li et al., 2020; Yan et al., 2018).

Dysregulation of Sirts, particularly Sirt1, Sirt2 and Sirt3, has been linked to mitochondrial dysfunction, oxidative stress, and neurodegeneration in PD. This suggests that Sirts could be promising therapeutic targets for PD (Liu et al., 2015). Experimental studies in PD models have reported that activation of the 5-HT_2A_ receptor by serotonin upregulates mitochondrial biogenesis and ATP production in cortical neurons via Sirt1 and PGC-1α (Fanibunda et al., 2019). The overexpression of Sirt1 has been reported to reduce αS aggregation and promote the survival of dopaminergic neuronal cells (Guo et al., 2016), while a small molecule Sirt1 agonist has been found to protect neurons from cell death in a PD animal model ((Lofrumento et al., 2014). On the other hand, the inhibition of Sirt2 has been reported to lower the concentration of toxic and submicroscopic αS oligomeric aggregates and reduce apoptosis in PD models (Outeiro et al., 2007). The upregulation of Sirt3 protects against impaired electron transport and ATP production, loss of mitochondrial membrane potential (MMP) and neuronal vulnerability, and treatment with Sirt3 agonists can restore mitochondrial integrity, reduce αS oligomers and oxidative stress. However, αS downregulates Sirt3 levels, which further impairs mitochondrial functions (Park et al., 2020; Xin and Lu, 2020).

Interestingly, similar Sirts dysregulation is observed in diabetes, where reduced Sirt1 and Sirt3 activity compromises mitochondrial function and insulin signaling via the PI3K-Akt-FoxO axis, suggesting a shared molecular mechanism linking metabolic dysfunction and neurodegeneration (Lu et al., 2023). These converging findings indicate that sirtuins are key regulators of neuronal and mitochondrial homeostasis in PD as their impaired activity elevate oxidative stress, ATP depletion, αS accumulation, and dopaminergic neuronal loss (Xu et al., 2023). In this regard, the ability of various phytochemicals to enhance sirtuin activity and restore dysregulated metabolic signaling further supports sirtuin modulation as a promising strategy for phytochemical-based therapeutic intervention in PD.

## 5 Type 2 diabetes and the role of insulin signaling in PD

Type 2 diabetes (T2DM) and PD are both age-associated diseases that have a high prevalence due to increased life expectancy. Currently, approximately 422 million people worldwide are struggling with diabetes (Park et al., 2020; Ntetsika et al., 2025). T2DM is a chronic metabolic disease associated with hyperglycemia due to insulin resistance (IR), and is increasingly recognized as a key factor in the onset and progression of PD. Indeed, individuals with T2DM face a higher risk of developing PD and often experience a more aggressive disease course with rapid progression and greater neuronal damage. This can be attributed to shared cellular mechanisms, including disrupted insulin signaling, which impairs neuronal health, synaptic function and mitochondrial activity (Cheong et al., 2020; Lurette et al., 2023; Zaltieri et al., 2015; Haque et al., 2022). Type 2 diabetes (T2DM), which accounts for approximately 90% of all diabetes cases is considered an important metabolic contributor to the pathogenesis of PD. T2DM contributes to PD pathogenesis via reduced PI3K-Akt activation, which leads to GSK3β hyperactivity, promoting αS aggregation. Concurrently, dysfunctional insulin signaling depletes cellular NAD^+^ levels, further diminishing sirtuin activity and exacerbating mitochondrial dysfunction (Zaltieri et al., 2015; Haque et al., 2022; La Vitola et al., 2024).

Hyperglycemia and IR are responsible for worsening αS aggregation and mitochondrial dysfunction, both of which contribute to the loss of dopamine-producing neurons. αS mainly impairs mitochondria by reducing MMP and ATP production, increasing fragmentation and oxidative stress. Altogether, this contributes to neuronal apoptosis or cell death (Zaltieri et al., 2015; Haque et al., 2022; La Vitola et al., 2024). Such effects also increase metabolic imbalances present in T2DM, elevating oxidative and nitrative stress and fueling αS toxicity. Additionally, these metabolic disturbances affect the Sirts. Under metabolic stress, Sirts activity may drastically decline, weakening the protective roles of these proteins and contributing to both mitochondrial dysfunction and protein imbalance. Metabolic stress in T2DM impairs the function of Sirt1 and Sirtt3, leading to decreased NAD^+^ availability. This results in impaired mitochondrial integrity, impaired defense against ROS and imbalances in protein homeostasis. Metabolic dysfunction, αS accumulation, mitochondrial damage and reduced Sirts activity form a complex pathological network linking T2DM and PD (Zaltieri et al., 2015; Haque et al., 2022; La Vitola et al., 2024). Moreover, the pathophysiology of T2DM, like PD, involves deleterious protein aggregation and cytotoxicity. In T2DM, the build-up of islet amyloid polypeptide (IAPP) has been linked to damaging effects on insulin-producing β cells of the pancreas (Yu et al., 2022; Park et al., 2020). GLP-1 and its binding receptor agonist stimulate insulin secretion in response to glucose levels and increase cellular sensitivity to insulin. This results in significant reduction in both systemic and brain insulin resistance.

**FIGURE 1:**
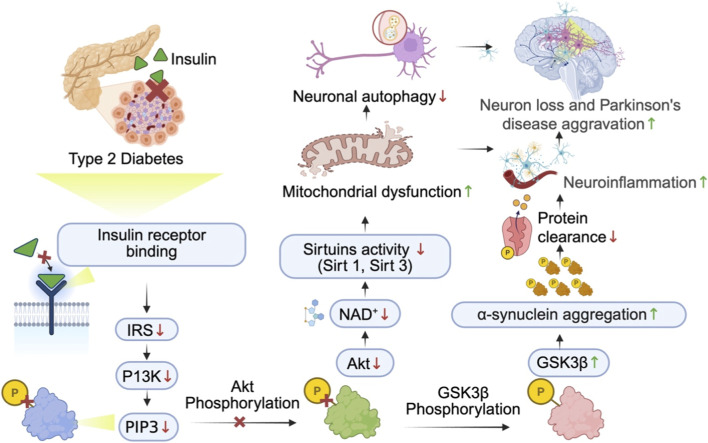
Proposed molecular mechanisms linking type 2 diabetes mellitus (T2DM) and Parkinson’s disease (PD) through impaired insulin signaling. T2DM is characterized by systemic insulin resistance and impaired insulin receptor signaling, which can extend to the central nervous system and disrupt neuronal insulin-dependent survival pathways. Reduced insulin receptor activation suppresses the IRS-PI3K-Akt cascade, leading to diminished Akt phosphorylation. Because Akt regulates mitochondrial homeostasis, autophagy, and inhibition of GSK3β, decreased Akt activity results in reduced NAD^+^ availability and Sirt1/Sirt3 function, mitochondrial dysfunction, and impaired neuronal autophagy. Concurrently, loss of Akt-mediated inhibition enhances GSK3β activity, promoting αS aggregation, defective protein clearance, and neuroinflammation. These converging metabolic and neurodegenerative disturbances may accelerate dopaminergic neuronal loss and aggravate PD pathology when T2DM and PD coexist. Arrows indicate directional relationships; ↑ denotes increased activity or levels; ↓ denotes decreased activity or levels; P denotes phosphorylation.

Insulin signaling in the brain primarily involves the PI3K/Akt/GSK3β signaling pathway, which is essential for neuronal survival, synaptic function and mitochondrial integrity. Whenever insulin is binding to its receptor, it triggers the phosphorylation of insulin receptor substrate (IRS) proteins, activating phosphatidylinositol 3-kinase (PI3K). PI3K then produce phosphatidylinositol (3,4,5)-trisphosphate (PIP3), which recruits and activates Akt (protein kinase B). The activated Akt further phosphorylates and inhibits the glycogen synthase kinase GSK3β (La Vitola et al., 2024; AlRuwaili et al., 2025; Yang et al., 2018). In PD, this pathway is often disrupted due to IR in the brain. This results in reduced Akt activation and uncontrolled GSK3β activity. This also promotes the buildup of αS, mitochondrial dysfunction, and increases neuronal loss. Altogether, this contributes to neuroinflammation, oxidative damage, and neuronal death, all of which are observed in PD (La Vitola et al., 2024; AlRuwaili et al., 2025; Yang et al., 2018; Soni et al., 2024; Ruiz-Pozo et al., 2023) The PI3K/Akt pathway is also responsible for regulating protein clearance systems like autophagy and the ubiquitin-proteasome system. When insulin signaling is disrupted, these systems can be compromised, allowing the accumulation of toxic proteins aggregates like αS and worsening neurodegeneration. Insulin signaling affects particularly Sirt1 and Sirt3. The latter heavily rely on NAD^+^ to support mitochondrial health, antioxidant defense, and metabolic balance. IR can disrupt this NAD^+^ metabolism, diminishing sirtuin activity and weakening mitochondrial function and neuronal cell survival (Figure 1) (Yang et al., 2018; Soni et al., 2024; Ruiz-Pozo et al., 2023).

## 6 GLP-1 and incretin mimetics in PD

The incretin hormones known as GLP-1 and GIP are released from enteroendocrine L-cells and K-cells in the small intestine in response to nutrient ingestion, where they play a key role in regulating glucose metabolism and maintaining systemic nutrient homeostasis (Müller et al., 2019; Müller et al., 2025). One of the earliest recognized actions of incretin hormones is the enhancement of glucose-dependent insulin secretion, which accounts for greater insulin release and lower glycemic responses following oral compared to intravenous glucose administration (Müller et al., 2019). Additional physiological roles in pancreatic islets, the brain, bone, and peripheral tissues have since been identified, and disturbances in incretin signaling are now considered to contribute to the pathogenesis of T2DM (Müller et al., 2019). The identification of exendin-4 from Gila monster *(Heloderma suspectum)* saliva as a stable GLP-1 mimetic enabled appetite suppression and glycemic control in metabolic disease, driving the development of long-acting GLP-1 analogues and unimolecular agents that co-activate GLP-1 and GIP receptors (Müller et al., 2025). These therapies are now widely utilized for the management of obesity, T2DM, and related complications (Hong et al., 2024).

Although direct involvement of GLP-1/GIP system abnormalities in PD remains uncertain, growing evidence shows that incretin-based therapies can improve cognitive function and exert neuroprotective effects, positioning them as promising candidates for treating neurodegenerative disorders (Müller et al., 2025). Furthermore, GLP-1 and GIP are mainly gut-derived peptide hormones released post-prandially that can improve glucose-dependent insulin secretion and maintain metabolic homeostasis (Hölscher, 2025). Beside their peripheral effects, GLP-1 and GIP receptors broadly found in PD affected key brain regions including substantia nigra, cortex, hippocampus, cerebellum, and associated glial populations (Hölscher, 2025; During et al., 2003). Interestingly, activation of these receptors can trigger intracellular signaling cascades; particularly cAMP/PKA/CREB. Under certain conditions, PI3K/Akt is also activated as it supports neuronal energy regulation, synaptic function, antioxidant defense, and autophagy (Müller et al., 2019; Hölscher, 2025; Baggio and Drucker, 2007; Doyle and Egan, 2007).

In PD models, Agonists of GLP-1 receptor have also been shown to restore impaired GLUT expression and stabilize glucose uptake in brain that supports improvement in metabolic flexibility (Hölscher, 2025; Duarte et al., 2020; Wang et al., 2023). Incretin receptor activation can also ameliorate insulin signaling by reducing aberrant IRS-1 phosphorylation and Akt activation in multiple PD experimental systems (Long-Smith et al., 2013). Furthermore, because Akt suppresses GSK3β, a kinase that drives neuronal apoptosis and αS pathology, GLP-1/GIP agonism can attenuate downstream toxic signaling events relevant to PD neurodegeneration (Hölscher, 2025; Yuan et al., 2025). Furthermore, incretin-based therapies can also improve mitochondrial function and reduce apoptosis by increasing anti-apoptotic protein Bcl-2 expression and lowering pro-apoptotic BAX signaling, thereby promoting neuronal survival in dopaminergic cell lines (Hölscher, 2025).

GLP-1 receptor agonists (e.g., exenatide, liraglutide, lixisenatide) and dual GLP-1/GIP agonists (e.g., tirzepatide) have been also reported to reduce microglial and astrocytic hyperactivation and suppress the release of inflammatory mediators such as TNF-α, IL-1β, and NF-κB. Additionally, these agents improve mitochondrial function and ATP production, oxidative stress and αS aggregation, and impaired insulin-signaling pathways (Huang et al., 2021; Aviles-Olmos et al., 2013; Lv et al., 2024). In addition to this, these agents also help in restoring the activity of Sirt1 and Sirt3 while elevating the NAD^+^ levels to maintain mitochondrial stability and overall metabolic balance within neurons (Aviles-Olmos et al., 2013). In toxin-induced and genetic PD models, treatment with GLP-1 mimetics such as exenatide or liraglutide have been shown to preserve dopaminergic neurons within the substantia nigra as well as improve motor function by restoration of dopamine levels in the striatum (Huang et al., 2021). Studies have also shown that GLP-1 receptor agonists (GLP-1RAs) can cross the blood-brain barrier (BBB) and act directly on neurons and glial cells to reduce the key pathological conditions in PD (Lv et al., 2024).

Clinical studies support these mechanistic effects. In a randomized, double-blind, placebo-controlled trial, once-weekly exenatide (2 mg) administered for 48 weeks significantly improved motor scores in patients with PD, with benefits sustained even after treatment discontinuation (Aviles-Olmos et al., 2013; Lv et al., 2024). More recently, the LixiPark phase 2 trial reported that lixisenatide significantly slowed the progression of motor disability compared with placebo in patients with early PD over 12 months (Athauda et al., 2017). However, a larger trial of the brain-penetrating PEGylated exendin-4 analog NLY01 did not meet its primary clinical endpoint, although secondary analyses suggested potential cognitive benefits (Meissner et al., 2024). Preclinical studies of dual GLP-1/GIP receptor agonists such as tirzepatide demonstrate synergistic actions, combining GLP-1–mediated anti-inflammatory effects with GIP-driven neurotrophic support, thereby enhancing dopaminergic neuron preservation and improving motor function (Meissner et al., 2024).

**FIGURE 2:**
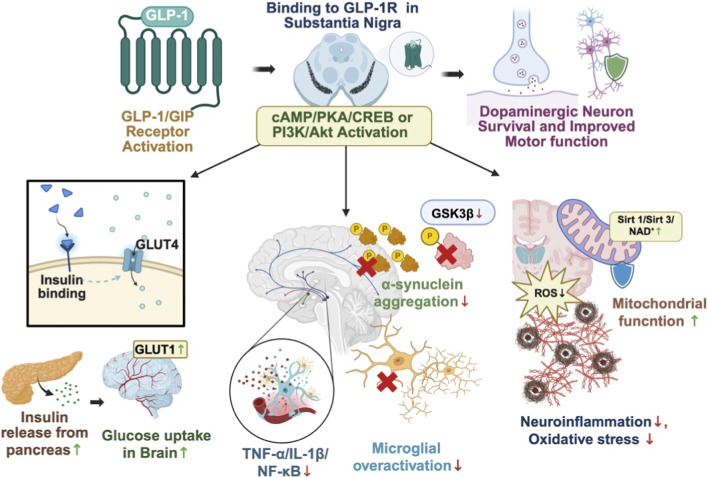
Neuroprotective and Metabolic Mechanisms of GLP-1/GIP Receptor Activation in Parkinson’s Disease. GLP-1/GIP receptor agonists bind to GLP-1 receptors expressed in the substantia nigra and activate downstream cAMP/PKA/CREB and PI3K/Akt signaling pathways. This signaling cascade improves dopaminergic neuron survival and motor function while simultaneously restoring metabolic and mitochondrial homeostasis. In the periphery and brain, GLP-1/GIP agonists enhance insulin sensitivity, promote GLUT1/GLUT4-mediated glucose uptake, and reduce insulin resistance. In the central nervous system, receptor activation inhibits GSK-3β activity, reduces αS aggregation, suppresses microglial overactivation and production of inflammatory mediators (TNF-α, IL-1β, NF-κB), and decreases ROS by improving mitochondrial function via increased Sirt1/Sirt3 and NAD^+^ levels. Collectively, these convergent effects mitigate neurodegenerative mechanisms in PD and support enhanced neuronal survival and function. The cross sign in figure denotes suppression or inhibition.

Collectively, these findings indicate that incretin mimetics provide neuroprotection extending beyond metabolic regulation by targeting multiple pathogenic processes in PD, including mitochondrial dysfunction, neuroinflammation from oxidative stress, sirtuin dysregulation, insulin resistance, and αS toxicity (Meissner et al., 2024). By promoting these neuroprotective pathways and improving insulin signaling, GLP-1–based therapies offer substantial protection against T2DM-related neuronal damage in PD (Müller et al., 2019). Taken together, GLP-1–based and dual GLP-1/GIP agonists represent a biologically plausible and clinically feasible strategy for repurposing as adjunctive neuroprotective therapies in PD, although further preclinical and clinical studies are needed to define their optimal dosing, treatment duration, and combinational approaches to ensure sustained neuroprotection (Figure 2).

## 7 PD and mitochondrial stress

Mitochondria are vital organelles that support cellular functions and cell survival. Their main function is ATP production. In doing so, they generate reactive oxygen species (ROS) as a by-product (Lee et al., 2010). Dysfunctional ATP production, due to ageing and ageing-related diseases, leads to increased ROS levels (Mathias et al., 2014; Li et al., 2021; Reczek and Chandel, 2015; Sakowska et al., 2015). In PD, the accumulation of αS in the SNpc leads to a downregulation of Sirt3 expression (Park et al., 2020). As Sirt3 regulates mitochondrial antioxidative enzymes, a deficiency in Sirt3 can aggravate dopaminergic neuron degeneration (Outeiro et al., 2007; Liu et al., 2015; Elgass et al., 2013). The accumulation of αS oligomers in PD promotes the phosphorylation of Dynamin-Related Protein 1 (Drp1), an important regulator of mitochondrial biogenesis (Reczek and Chandel, 2015). This, in turn, stimulates mitochondrial fission and increases mitochondrial stress. αS also interacts and inhibits the activity of TOM20 (Di Maio et al., 2016), which impairs the redistribution of Sirt3 into mitochondria from the cytoplasm and increases mitochondrial stress (Motyl et al., 2018). Moreover, extracellular αS has been shown to transcriptionally regulate Sirt family genes, leading to reduced mitochondrial membrane potential and decreased cell viability (Motyl et al., 2018).

## 8 PD and mitophagy

Mitophagy, also known as mitochondrial autophagy, is a type of autophagy, a self-regulatory process used by cells to degrade ectopic and/or aggregated proteins and impaired organelles (Lee et al., 2023). PD has been linked with mitophagy dysfunction via different molecular mechanisms. Whilst primarily located in the cytosol, in PD abnormal αS may translocate into mitochondria. Activation or overexpression of Sirt3 can degrade accumulated αS oligomers by regulating mitophagy (Yu et al., 2022; Park et al., 2020). Aggravated accumulation of αS disrupts mitochondrial biogenesis and function by dysregulating the AMPK-CREB-PGC-1α pathway (Shi et al., 2005), which regulates Sirt3 and mitochondrial defence (Park et al., 2020), reported that restoration of Sirt3 expression through the AMPK agonist 5-aminoimidazole-4-carboxamide riboside (AICAR) could recover mitochondrial dynamics and mitophagy. Furthermore, pharmacological inhibition of autophagy suppresses Sirt3 activity, resulting in αS accumulation and enhanced autosis, an autophagy-dependent form of cell death (He et al., 2022).

In the MPP^+^-induced PD model, Sirts were found to be degraded primarily through autophagy rather than the proteasome (Baeken et al., 2021). Endogenous ROS, in the form of H_2_O_2_ or its decomposed products, oxidizes Sirts, which co-localise with autophagosomal structures and are degraded via autophagy. Since ROS promotes Sirts degradation in PD (Baeken et al., 2021)., Sirts-based therapeutic approaches should not only aim to increase Sirts expression but also focus on reducing the stress to maintain Sirts bioavailability and cellular homeostasis.

Cysteine residue oxidation of the inner mitochondrial protein MIC60 triggers disulphide bonding with Miro, an outer mitochondrial membrane protein (Gupta et al., 2022). This structural alteration delays the cellular response to mitophagy and disturbs normal mitochondrial function. The Miro protein plays a crucial role in mitochondrial turnover through PINK1/Parkin-mediated mitophagy. Miro1 possesses multiple lysine residues that undergo ubiquitination, and impairment of Miro1 ubiquitination hinders the translocation of E3 ubiquitin ligase Parkin (López-Doménech et al., 2021), leading to delayed mitophagy in response to damaged mitochondria (Gupta et al., 2022; Doyle and Egan, 2007).

## 9 PD and apoptosis

αS-mediated toxicity is considered the key factor for neuronal loss in PD pathogenesis, although, the molecular mechanism underlying this process is still unclear.

Sirts can protect against αS protein aggregation. Rotenone treated *in vitro* PD model showed impairment of lysosomal functions due to decreased NAD^+^/NADH and Sirt1 expression (Dong W et al., 2021). Although, the exact mechanism of the Sirt2 inhibitor’s effect on αS aggregation remains unclear, however, a study showed that Sirt2 inhibition increases acetylation of α-tubulin (Outeiro et al., 2007). Since increased α-tubulin acetylation has been reported to interact with αS and stabilises microtubules (Jensen et al., 2000), it could be possible that increased acetylated α-tubulin stimulates αS aggregations through its affinity to microtubules (Outeiro et al., 2007) and promotes cell death. As a presynaptic protein αS can easily convert into different conformations, depending on the stress.

In a study, extracellular αS has been found to upregulate Sirt3 and Sirt5 but downregulate Sirt1 (Motyl et al., 2018) which plays important role in cell metabolism. Extracellular αS has also been shown to regulate transcription factors of Sirts and the upregulation of αS increases pro-apoptotic Bax-mediated cell death (Motyl et al., 2018). The upregulation of Sirt3 and Sirt5 may have anti-apoptotic activities (Kincaid and Bossy-Wetzel, 2013), but decreased expression of Sirt1 disrupts the Sirt1-PGC-1α inter-relationships (Lavu et al., 2008). This disruption negatively affects DNA repair mechanisms and mitochondrial biogenesis, resulting in apoptotic cell death.

**FIGURE 3:**
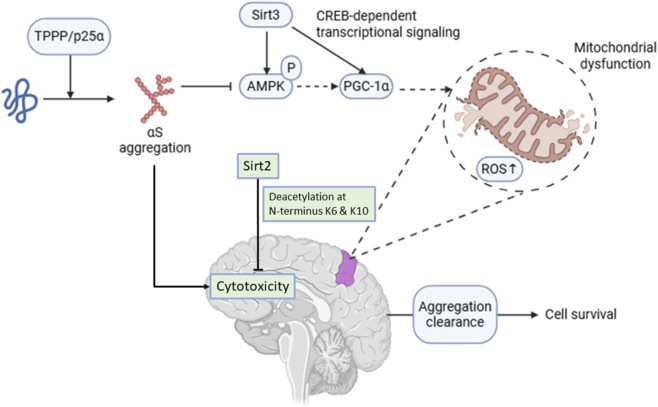
Sirtuin subtype-specific regulation of α-synuclein (αS) aggregation and mitochondrial dysfunction in Parkinson’s disease. Decreased Sirt3 activity impairs AMPK phosphorylation and downstream CREB/PGC-1α dependent transcriptional signaling, resulting in compromised mitochondrial bioenergetics, altered mitochondrial dynamics, and increased reactive oxygen species (ROS) production, which contribute to neuronal vulnerability and degeneration. Co-expression of tubulin polymerization promoting protein (TPPP/p25α) and αS promotes the formation of cytotoxic oligomeric intermediates and fibrillar aggregates, enhancing cellular toxicity. Sirt2 regulates αS acetylation at N-terminal lysine residues (K6 and K10); inhibition of Sirt2 promotes microtubule stabilization via increased acetylation and facilitates sequestration of toxic αS species into juxtanuclear inclusions, thereby enhancing aggregate clearance and supporting cell survival.

Pharmacological activation and inhibition of Sirts have shown protective effects against apoptotic cell death in PD models. For instance, activation of Sirt1 reduced toxin-induced neuronal death, whereas inhibition of Sirt2 ameliorated αS-mediated cell death in PD models (Figure 3) (Park et al., 2020). Several shreds of evidence suggest that Sirt3 activation could prevent αS-mediated dopaminergic neuronal loss by enhancing mitochondrial biogenesis and ROS (Gleave et al., 2017). However, in PD pathogenesis, oligomerisation of αS downregulates Sirt3 (Park et al., 2020), resulting in decreased phosphorylation of AMPK and increased phosphorylation of Drp1, impairing mitochondrial respiration, and leading to apoptosis.

Glial cytoplasmic inclusion of αS is the pathological hallmark of multiple system atrophy. Both oligomeric and monomeric conformation of αS accumulates in different brain regions including putamen, substantia nigra, and white matter of the internal capsule during disease pathogenesis. The accumulation is accompanied by the phosphorylation of αS oligomers at Ser-129 that triggers apoptosis via rapid retraction of microtubules from the cellular process (Kragh et al., 2009). Specific inhibition of Sirt2 suppressed the apoptotic phenomenon and increased number of insoluble αS positive inclusions (Outeiro et al., 2007; Kragh et al., 2009). Sirt2 inhibition increases α-tubulin acetylation that stabilises the microtubular network and αS aggregates transportation (Sanders and Greenamyre, 2013). Thus, by stabilising the microtubule network, the cytotoxicity of intermediate αS fibrils or oligomers could be reduced and, the degradation of aggregated proteins could be improved in synucleinopathies.

## 10 PD and neuroinflammation

Microglial cells in the brain are highly sensitive to surrounding microenvironmental changes and cellular damage due to ROS-induced apoptotic neuronal cell death (Sanders and Greenamyre, 2013). In PD, αS accumulation, inflammatory response, microglial activation and neuronal damage are all interlinked. The presence of αS aggregates in PD can directly activate microglia. The latter can also be activated as a result of apoptotic neuronal death (Hoffmann et al., 2016; Jiang et al., 2015). As activated microglia attempt to clear αS aggregates, this leads to the release of proinflammatory mediators and ultimately neurotoxicity (Hoenen et al., 2016).

The microglial inflammasome describes a unique innate immune system that regulates the inflammatory response in the brain (Hoenen et al., 2016; Tyagi et al., 2018). The release of proinflammatory mediators by microglia is typically mediated by the NF-κB pathway. Post-translational modification and nuclear localisation of NF-κB is a key event in the regulation of the transcription and expression of target genes, including those coding for interleukins (IL1b, IL18). NF-κB within activated microglia also promotes inducible nitric oxide synthase (iNOS) that produces nitric oxide (NO) which in turn enhances αS aggregation by impairing the ubiquitin-proteasome machinery (Tao et al., 2019). Interestingly, nitrosative modifications to αS within Lewy bodies have been observed in postmortem PD brains (Gu et al., 2005; Rocha et al., 2018).

**FIGURE 4:**
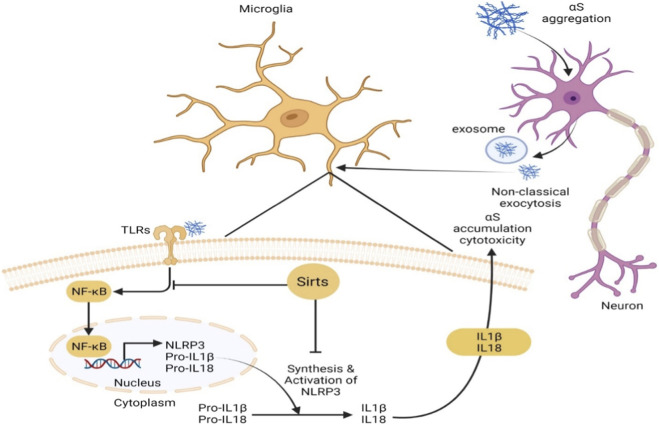
αS provokes NLRP3 inflammasome activation and cytotoxicity. The imbalance between αS synthesis and clearance and/or intrinsic and extrinsic factors contributes to αS accumulation in neurons. Neurons release αS aggregates via distinct active and/or passive mechanisms; exosomes are involved in this releasing process. αS aggregates at the extracellular space provide a priming signal for the activation of NLRP3 through binding to TLRs, triggering NF-κB-dependent upregulation of NLRP3 and production of pro-inflammatory cytokines (pro-IL-1β and pro-IL-18). In addition, αS aggregates impair mitochondrial function following the internalisation of α-syn fibrils by microglia, which includes the reduction of Sirt3 via the AMPKα-CREB signalling pathway, the blockage of TOM20 and the engagement of CD11b. The activation of the NLRP3 inflammasome that induces activated caspase-1-mediated release of mature IL-1β and IL-18, is also capable of augmenting cytotoxicity and αS accumulation. Several therapeutic approaches (e.g., antibodies, drugs and natural extracts) can exert neuroprotection by targeting αS/TLRs/NF-κB/NLRP3.

Sirts are involved in the regulation of the microglial inflammatory response. For example, the knockdown of Sirt3 or silencing of Sirt4 has been found to increase IL-1β levels via microglial hyperactivation (Jiang et al., 2015; Tyagi et al., 2018). Such Sirts have the potential to downregulate the NF-κB pathway, which helps reduce neuroinflammation (Paxinou et al., 2001) Sirt2 has been reported to deacetylate NOD-like receptor pyrin domain containing 3 (NLRP3) and prevent aging-related chronic inflammation (Li et al., 2021), while Sirt3 inhibits NLRP3 by reducing mitochondrial damage and mtDNA release (He et al., 2020). Sirt2 inhibition reduces histone hypoacetylation in dopaminergic neurons and downregulates neuroinflammation via reducing iNOS and COX-2 expression (Harrison et al., 2018). Since αS and its familial mutants (A53T and A30P) inhibit histone acetylation at the chromatin level by masking H3 (Kontopoulos et al., 2006; Jamali-Raeufy et al., 2019), Sirt2 inhibitors have shown substantial benefit in PD therapy (Gleave et al., 2017) via downregulation of αS toxicity (Qin et al., 2017). The effects of αS and Sirts on the microglial inflammasome has been reviewed elsewhere (Giasson et al., 2000; Haque et al., 2020). There are summarized in Figure 4.

## 11 The role of phytomolecules as an emerging candidate for PD

In addition to incretin-mediated effects, several plant-derived phytochemicals target innate immune receptors and inflammatory signaling cascades that contribute to chronic neuroinflammation in both PD and AD (Balakrishnan et al., 2021). Activation of TLRs on microglia by misfolded proteins (such as αS) can trigger downstream NF-κB signaling and amplify pro-inflammatory cytokine secretion (Fiebich et al., 2018). Plant-derived metabolites like andrographolide have been shown to decrease microglial activation and inhibit α-synuclein induced neurotoxicity. They further suppress the NF-κB/NLRP3 pathway, caspase-1 activity and IL-1β production, and attenuate dopaminergic neuron loss in PD models (Ahmed et al., 2021).

The NLRP3 inflammasome comprises complex signaling molecules that contribute to chronic inflammation in neurodegenerative disorders. Several phytochemicals, including berberine and other isoquinoline alkaloids, inhibit the assembly and activation of NLRP3, reducing mature pro-inflammatory cytokine release and mitigating neuroinflammation and neuronal loss in PD models (Anderson et al., 2023).

Beyond immunomodulation, many plant phytoconstituents exert antioxidant and mitochondrial protective effects that are crucial in neurodegeneration. By scavenging reactive oxygen species (ROS) and enhancing endogenous antioxidant defenses, compounds such as curcumin, resveratrol, quercetin and EGCG activate cytoprotective pathways like Nrf2/ARE, improve mitochondrial function, and reduce oxidative stress-induced apoptosis in neuronal cells (Ahmed et al., 2026).

**FIGURE 5:**
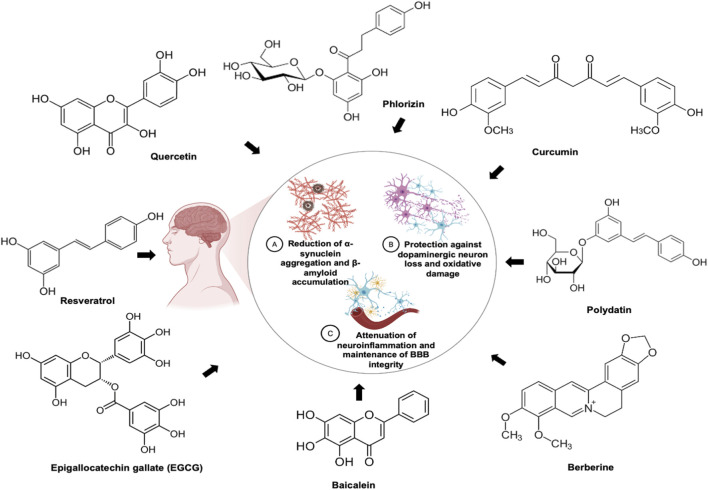
Common neuroprotective mechanisms of phytomolecules showing activity in models of Parkinson’s disease (PD). Phytoconstituents such as resveratrol, curcumin, epigallocatechin gallate (EGCG), quercetin, Phlorizin, baicalein, polydatin and berberine demonstrate neuroprotective effects in PD by reducing αS aggregation, protecting dopaminergic neurons from oxidative and mitochondrial damage, and alleviating neuroinflammation while preserving the blood–brain barrier (BBB) integrity.

Current therapeutic options for PD such as levodopa/carbidopa, pramipexole, selegiline, entacapone, trihexyphenidyl and deep brain stimulation are often limited by high cost, restricted accessibility and significant adverse effects including hallucinations, impulse-control disorders, dyskinesias, hepatotoxicity and cognitive impairment as well as surgery-related complications. Importantly, these interventions primarily offer symptomatic relief while failing to address the underlying disease-modifying mechanisms (Zahoor et al., 2018). None of these therapeutic interventions reliably halt or reverse the underlying neurodegenerative progression in PD. Significant gaps remain, particularly the lack of treatments with adequate safety and true disease-modifying potential capable of addressing key pathological drivers such as neuroinflammation, mitochondrial dysfunction, impaired proteostasis and toxic protein aggregation (Jiang et al., 2025). Therefore, there is an urgent need for alternative therapeutic approaches, including plant-derived phytochemicals, that can directly modulate the core pathogenic mechanisms of PD, such as neuroinflammation, mitochondrial dysfunction and αS aggregation. Several naturally occurring compounds have shown promising neuroprotective effects in preclinical models of PD. Notable examples include resveratrol (*Polygonum cuspidatum*), curcumin (*Curcuma longa*), epigallocatechin gallate (EGCG; *Camellia sinensis*), quercetin (*Allium cepa*), phlorizin (*Malus domestica*), baicalein (*Scutellaria baicalensis*) and berberine (*Berberis* spp.) (Figure 5) (Haque et al., 2020; Balakrishnan et al., 2021; Naik et al., 2025).

Curcumin and resveratrol inhibit NF-κB activation and suppress the transcription of NLRP3 inflammasome components and pro-inflammatory cytokines (IL-1β, IL-18) (Gonzales and Orlando, 2008). Epigallocatechin gallate (EGCG) attenuates TLR-mediated signaling, decreasing microglial activation triggered by extracellular αS aggregates (Zhong et al., 2019). Quercetin and other polyphenols inhibit NLRP3 inflammasome assembly, thereby reducing caspase-1 activation and limiting the release of (IL-1β, IL-18), this results in a significant reduction in neuroinflammatory cytotoxicity (Xue et al., 2017). Baicalein inhibits αS aggregate formation and suppresses the NF-κB/NLRP3 signaling pathway (Rui et al., 2020). Berberine mitigates microglial activation and promotes neuroprotection by blocking the TLR4/NF-kB pathway (Tian et al., 2023). Oral phlorizin treatment counteracted dopamine depletion, alleviated neuroinflammatory responses, and rescued motor deficits in a PD mouse model (Zhang G. et al., 2019).

Resveratrol from *Polygonum cuspidatum*, a phytoconstituent which belongs to the stilbene class of natural products. Resveratrol exerts neuroprotection in PD by reducing ROS, and preventing the apoptosis of dopaminergic neurons. These effects are partly mediated through the activation of Sirts and improved mitochondrial function. The benefits of resveratrol also extend to T2DM, where it helps regulate glucose metabolism and insulin sensitivity (Olszowy-Tomczyk and Wianowska, 2024; Wang et al., 2011). In transgenic animal models of PD, treatment with resveratrol improves motor and cognitive functions, decreases neuroinflammation, and directly prevents αS aggregation (Zhang et al., 2018). Resveratrol also promotes mitochondrial biogenesis by reducing PGC-1α acetylation (Kumar et al., 2006). It was shown to trigger the LC3 deacetylation-mediated autophagic degradation of αS via Sirt1 activation in PD mice models (Guo et al., 2016). Polydatin is another stilbene derivative from *P. cuspidatum* that has also been reported to improve motor symptoms and boost mitochondrial function in PD animal models, in part by activating Sirt1 (Malar et al., 2020; Zhang X. Y. et al., 2020).

The diarylheptanoid curcumin, a polyphenolic compound derived from *Curcuma longa*, has demonstrated protective effects in neurodegenerative and metabolic disorders through modulation of key signaling pathways involved in oxidative stress, apoptosis, inflammation, and cell survival. In models of PD, curcumin activates the PI3K/Akt signaling pathway, which is crucial for promoting neuronal survival, enhancing neurotrophic support and reducing inflammation and oxidative damage, thereby improving dopaminergic neuron integrity and motor behavior (Das et al., 2023; Song et al., 2021; Ren et al., 2020). Experimental studies have also demonstrated that curcumin’s neuroprotective efficacy in PD is associated with activation of PI3K/Akt, reduction of pro-oxidative stress markers and preservation of neuronal function, underpinning its role in delaying disease progression (Nebrisi, 2021). Additionally, curcumin modulates Sirt1-Foxo1 signaling and enhances PI3K-Akt activity, leading to restored Akt phosphorylation, decreased pro-apoptotic signaling, and improved cellular antioxidant defenses. Collectively, these mechanisms encompassing PI3K/Akt activation, modulation of Sirt1-Foxo1, reduction of oxidative stress, inhibition of apoptosis, and cholinergic receptor-mediated anti-inflammatory effects, provide a robust biological basis for curcumin’s neuroprotective and cytoprotective effects in PD (Nebrisi, 2021).

EGCG from green tea (*Camelia sinensis*) can reduce αS aggregation and the loss of dopamine by inhibiting MAO-B and COMT. It also enhances antioxidant defenses via chelating iron. This contributes to minimizing neurodegeneration in PD. It has also been shown to increase insulin sensitivity in T2DM (Malar et al., 2020; Liu et al., 2023). Beyond neuroprotection, EGCG improves insulin sensitivity and glucose homeostasis in T2DM by enhancing insulin receptor signaling, reducing insulin resistance, and modulating metabolic pathways in skeletal muscle and liver, ultimately lowering fasting glucose and improving β-cell function in experimental models. These combined neuroprotective and metabolic effects support EGCG’s potential as a multifaceted phytochemical agent capable of attenuating both neurodegenerative and metabolic dysfunctions (Caruana and Vassallo, 2015; Yurtseven and Yücecan, 2024).

Quercetin, a flavonoid from *Allium cepa* (onion) and *Malus domestica* (apple), can activate Sirts, particularly Sirt1, reducing mitochondrial ROS and dysfunction. It has also been reported to inhibit αS aggregation and cytotoxicity in PD animal models, promoting neuronal cell survival (Xue et al., 2017; Rui et al., 2020; Das et al., 2023). Through this Sirt1 modulation, quercetin promotes mitochondrial quality control and mitophagy via the PINK1-Parkin pathway, thereby preserving mitochondrial integrity and preventing dopaminergic neuron loss in PD models. In addition to reducing oxidative stress, quercetin has been reported to directly inhibit αS aggregation and associated cytotoxicity, helping to maintain neuronal cell survival and attenuate key pathological hallmarks of PD. It also suppresses neuroinflammation by downregulating NF-κB and inflammasome pathways and enhances antioxidant defenses by increasing Nrf2 signaling and expression of endogenous antioxidant enzymes (Zhu et al., 2013; Cui et al., 2022).

Baicalein, a flavonoid from *Scutellaria baicalensis*, has shown neuroprotective effects in multiple PD models by suppressing neuroinflammation and reducing αS aggregation. It can restore cell viability, decrease ROS and lipid peroxidation, and minimise dopaminergic neuronal loss and motor deficits (Song et al., 2021). Baicalein can modulate iron homeostasis in PD animal models by protecting aconitase 1 (ACO1) from oxidative inactivation and inhibiting IRP1 activation, thereby limiting brain iron accumulation (Liu et al., 2023). Baicalein also inhibits the activation of the NLRP3 inflammasome and downstream caspase-1/GSDMD signaling, overall reducing the release of neuroinflammatory mediators and mitigating neuronal damage (Zahoor et al., 2018; Rui et al., 2020; Balakrishnan et al., 2021; Jiang et al., 2025).

Berberine, an isoquinoline alkaloid from *Berberis* spp., has demonstrated promising antioxidant activity via promoting heme oxygenase-1 (HO-1) expression, activating Nrf2/AMPK/SIRT1 signaling, inhibiting the NLRP3 inflammasome, balancing glutathione levels, and regulating mitochondrial redox processes (Sunhe et al., 2024; Huang et al., 2021). In dopaminergic toxin-induced PD models, berberine has been reported to minimize neuronal loss, improve motor function, mitochondrial respiration, and decrease caspase-3 activation (Cha et al., 2025).

Phlorizin, found in *M. domestica,* primarily functions as a natural sodium–glucose cotransporter (SGLT1/2) inhibitor, improving systemic glucose homeostasis and insulin sensitivity, which helps counteract PD-associated insulin resistance. Additionally, by inhibiting DPP-IV, phlorizin prolongs endogenous GLP-1 action, enhancing neuroprotective PI3K/Akt and cAMP/PKA/CREB signaling and reducing neuronal apoptosis. Beyond its metabolic benefits, phlorizin reduces ROS, activates AMPK-dependent mitochondrial protection, and attenuates neuroinflammation. Through these combined mechanisms, phlorizin helps preserve dopaminergic neuron integrity and reduces vulnerability to metabolic and inflammatory stressors in PD (Singh et al., 2020; Zhang X. Y. et al., 2020; Ni et al., 2024).

Several plant-derived phytochemicals have been shown to influence the incretin system, specifically by modulating dipeptidyl peptidase-4 (DPP-IV) activity and thereby prolonging the half-life of GLP-1, GIP, and other incretin hormones. For instance, quercetin (from *A. cepa* and *M. domestica*) exhibits significant DPP-IV inhibitory activity *in vitro* (Singh et al., 2020). In addition, resveratrol and curcumin have also been shown to stimulate GLP-1 secretion and/or enhance incretin receptor responsiveness, thereby improving insulin sensitivity and metabolic homeostasis in diabetes models (Dao et al., 2011; Zhu et al., 2017; Alli-Oluwafuyi et al., 2019). In the context of PD, incretin signalling particularly via GLP-1 or dual GLP-1/GIP receptor agonism has demonstrated direct neuroprotective effects, including improved mitochondrial function, reduced neuroinflammation, preservation of dopaminergic neurons, and decreased αS aggregation (Dao et al., 2011; Zhu et al., 2017; Alli-Oluwafuyi et al., 2019; Nowell et al., 2023). These actions are mediated through enhanced mitochondrial biogenesis and ATP production, inhibition of inflammatory signaling, and preservation of nigrostriatal dopaminergic neurons, collectively leading to improved motor and non-motor functional outcomes (Zhu et al., 2017; Alli-Oluwafuyi et al., 2019; Nowell et al., 2023).

Although direct evidence connecting phytochemicals to incretin-mediated neuroprotection in PD is still emerging, it is biologically plausible that phytochemical-driven DPP-IV inhibition or incretin stimulation could enhance neuronal energy metabolism, mitochondrial function, and anti-inflammatory signaling, thereby protecting dopaminergic neurons. While phytochemical effects on incretin pathways are mainly supported by metabolic and diabetes studies, and incretin-mediated neuroprotection in PD is well-documented, combining these findings provides a strong rationale for exploring plant-derived compounds as adjunctive neuroprotective strategies in PD (Nowell et al., 2023).

Table 1 represents the current and emerging drug candidates and their molecular targets in PD. Table 2 summarize well-studied phytomolecules according to their phytochemical classes, botanical origins, pharmacological effects and mechanisms of action in PD.

**TABLE 1:** Current and emerging therapeutic agents targeting molecular mechanisms in Parkinson’s disease.

Category	Drug/Compound	Mechanism of action	Therapeutic effect	References
Current standard therapies	Pramipexole, ropinirole	Dopamine receptor	Stimulates D2/D3 receptors to reduce motor fluctuations	Sárkány et al. (2025)
	Entacapone, tolcapone	COMT inhibitors	Extend levodopa half-life and efficacy	Contin et al. (2019)
	Levodopa + carbidopa	Dopamine precursor + peripheral decarboxylase inhibitors	Restores dopamine levels and improves motor symptoms	Parkinson Study Group (1989)
	Selegiline	MA-O, B inhibitors	Prevent dopamine breakdown and reduce ROS.	Lees (2008)
	Amantadine	NMDA receptor antagonist	Reduces dyskinesia and glutamate-mediated toxicity	Butzer et al. (1975)
	Rasagiline	MA-O, B inhibitors	Prevent dopamine breakdown and reduce oxidative stress	Malaty and Fernandez (2009)
	Exenatide Lixisenatide Semaglutide	GLP-1 receptor agonist; NLRP3 inhibitors; antioxidative	Increase mitochondrial function and reduce neuroinflammation and αS aggregation, promotes mitochondrial function	Athauda et al., 2017, Zhang et al., 2019b
Emerging candidate drugs	Baicalein	NF-kB/NLRP3 inhibition	Reduction of αS aggregation and neuroinflammation	Rui et al. (2020)
	Curcumin	NLRP3/NF-kB suppression, antioxidant	Attenuates microglial, protects dopaminergic neurons	Benameur et al. (2023)
	Berberine	TLR4/NF-kB inhibition, mitochondrial protection	Enhances neuronal activity and reduces inflammation	Wang et al. (2011)
	Resveratrol	SIRT1 activation, antioxidative	Promotes mitochondrial function	Zhang et al. (2018)
	Phlorizin	SGLT/DPP-IV inhibition; ↑GLP-1	Protects dopaminergic neurons, reduces neuroinflammation	Ni et al., 2024, Zhang et al., 2020a

**TABLE 2:** Summary of phytomolecules with neuroprotective potential in Parkinson’s disease.

Phytomolecules	Phytochemical class	Botanical source	Plant part used	Major pharmacological effects in PD	Mechanistic targets/Pathways	References
Resveratrol	Stilbene	*Polygonum cuspidatum* (Japanese knotweed)	Root, rhizome	Reduces ROS, αS aggregation, improves mitochondrial function, and reduces neuroinflammation	↑SIRT1/PGC-1α; ↑mitochondrial biogenesis; ↑AMPK activity; ↑GLP-1 secretion; ↓NF-kB signaling	Kumar et al., 2006; Guo et al., 2016; Zhang et al., 2020a; Ren et al., 2020; Nebrisi, 2021
Polydatin	Stilbene glycoside	*Polygonum cuspidatum*	Root	Improves motor symptoms and mitochondrial function in PD models	↑ SIRT1 expression; and ↑mitochondrial biogenesis; ↓ROS production; ↑insulin sensitivity	Malar et al. (2020)
Curcumin	Diarylheptanoid	*Curcuma longa* (turmeric)	Rhizome	Reduces mitochondrial ROS, inflammation, and apoptosis; protects against PD and diabetic cardiomyopathy	↑SIRT1-FoxO1 and PI3K-Akt pathways; ↑activates α7-nAChR signaling; ↑insulin sensitivity; ↓NF-kB and DPP-IV	Das et al., 2023; Song et al., 2021
Epigallocatechin gallate (EGCG)	Flavonoid (catechin)	*Camellia sinensis* (green tea)	Leaf	Inhibits αS aggregation, enhances antioxidant defenses, chelates iron, and increases insulin sensitivity	↓MAO-B and COMT; ↑ antioxidant enzymes; ↓TLR/NF-kB signaling; ↑GLP-1 release	Liu et al. (2023)
Quercetin	Flavonol	*Allium cepa* (onion), *Malus domestica* (apple)	Bulb, fruit	Reduces mitochondrial ROS, inhibits αS aggregation, improves neuronal survival	↑SIRT1 and Nrf2 activation; reduces mitochondrial dysfunction and αS cytotoxicity; ↑insulin receptor sensitivity	Rui et al. (2020)
Baicalein	Flavonoid	*Scutellaria baicalensis* (Chinese skullcap)	Root	Attenuates oxidative stress, neuroinflammation, and αS aggregation; regulates iron homeostasis	↓NLRP3-caspase-1/GSDMD signaling; modulates ACO1/IRP1 axis; ↓ROS; ↑AMPK activation; ↑insulin responsiveness	Balakrishnan et al., 2021; Zahoor et al., 2018; Jiang et al., 2025
Berberine	Isoquinoline alkaloid	*Berberis* spp.	Root, bark	Reduces oxidative stress, improves mitochondrial respiration, suppresses neuroinflammation, and enhances antioxidant response	Activates Nrf2/AMPK/SIRT1 pathways; inhibits NLRP3 inflammasome; upregulates HO-1; ↑insulin sensitivity; ↓TLR4/NLRP3/NF-kB signaling	Sunhe et al., 2024; Huang et al., 2021; Cha et al., 2025
Phlorizin	Flavonoid	*Malus domestica* (apple)	Bark	Improves insulin sensitivity; enhances glucose metabolism; neuroprotection in PD	↓TLR4/NLRP3/NF-κB signaling; ↑AMPK activation; ↑GLP-1 action (via DPP-IV inhibition); ↑PI3K/Akt and cAMP/PKA/CREB pathway	Ni et al., 2024; Zhang et al., 2020a; Singh et al., 2020

**FIGURE 6:**
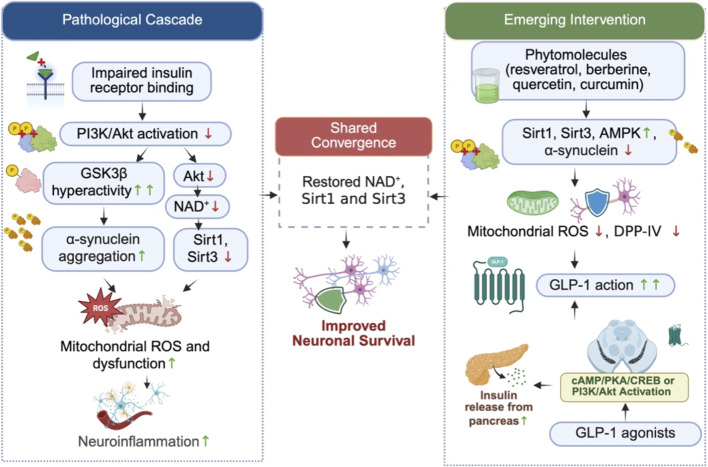
Integrated mechanistic framework illustrating the pathological cascade and emerging therapeutic interventions targeting the αS-Sirtuin-Insulin signaling axis in Parkinson’s disease. Left panel: Impaired insulin receptor binding reduces PI3K/Akt activation, leading to GSK3β hyperactivity and α-synuclein aggregation. Concurrently, reduced Akt activity depletes NAD+, downregulating Sirt1 and Sirt3, resulting in mitochondrial ROS, dysfunction, and neuroinflammation. Right panel: Phytomolecules including resveratrol, berberine, quercetin, and curcumin activate Sirt1, Sirt3, and AMPK, reducing α-synuclein aggregation and mitochondrial ROS. DPP-IV inhibition by phlorizin and quercetin prolongs endogenous GLP-1 action. GLP-1 agonists activate cAMP/PKA/CREB and PI3K/Akt signaling, restoring Sirt1/Sirt3 activity and improving motor function. Center: Both therapeutic strategies converge on restoring NAD+, Sirt1, and Sirt3 activity, collectively improving neuronal survival. ↑ denotes increased activity or levels; ↓ denotes decreased activity or levels.

Despite structural diversity, the phytochemicals often exhibit a common metabolic neuroprotective axis that intersects insulin signaling, sirtuin regulation, and αS pathophysioloy. Compounds such as resveratrol, curcumin, quercetin, EGCG, berberine, baicalein, polydatin, and phlorizin activate AMPK-SIRT1/PGC-1α and PI3K-Akt pathways, enhance mitochondrial biogenesis and bioenergetics, suppress NF-κB/NLRP3 driven neuroinflammation, reduce ROS production, and improve insulin responsiveness. Through these coordinated effects, they attenuate αS aggregation, promote proteostatic clearance, preserve dopaminergic neuron integrity, and maintain blood brain barrier stability. Thus, phytomolecules can intervene at multiple nodes of the insulin-sirtuin-mitochondrial-α synuclein network underlying PD neurodegeneration (Figure 6; Table 2).

## 12 Concluding remarks and future research

In summary, metabolic dysfunction in T2DM affects insulin receptor signaling, inhibiting the PI3K-Akt cascade and enhancing GSK3β hyperactivity, NAD^+^ depletion, and Sirt1/Sirt3 inactivation. These events collectively result in mitochondrial dysfunction, elevated ROS, and impaired protein clearance, the triad that drives αS misfolding and aggregation. Aggregated αS in turn reciprocally worsens mitochondrial stress and sirtuin loss, locking the system into a degenerative cycle that culminates in dopaminergic neuronal loss. Phytomolecules interrupt this cycle at multiple nodes simultaneously: activating AMPK-Sirt1/Sirt3-PGC-1α to restore mitochondrial biogenesis and NAD^+^ levels; reactivating PI3K-Akt to suppress GSK3βdriven αS aggregation; inhibiting NF-κB/NLRP3 neuroinflammation; and, via DPP-IV inhibition or direct GLP-1 stimulation, engaging incretin-mediated neuroprotective signaling. Thus, the insulin-sirtuin-mitochondrial-αS network constitutes a shared pathogenic axis linking T2DM and PD, and phytomolecules represent mechanistically rational, multi-target candidates for disease-modifying intervention at this convergence point.

Numerous studies have demonstrated the therapeutic potential of phytomolecules in preclinical cellular and animal PD models, with their ability to exert pleiotropic neuroprotective effects by targeting, inflammatory signaling, mitochondrial dysfunction, αS aggregation, and key pathways including sirtuins and PI3K/Akt signaling (Gonzales and Orlando, 2008; Zhong et al., 2019; Xue et al., 2017; Song et al., 2021; Liu et al., 2023; Rui et al., 2020; Sunhe et al., 2024; Huang et al., 2021, Cha et al., 2025; Bhusal et al.,2023; Shahpiri et al., 2016; Niu et al., 2025). However, despite these promising findings, definitive clinical evidence remains limited. Most clinical investigations conducted to date involve small pilot or open-label studies, highlighting a pressing need for larger, rigorously controlled trials to establish optimal dosing, treatment duration, and therapeutic efficacy in patients (Balakrishnan et al., 2021; Shahpiri et al., 2016; Niu et al., 2025).

Future research should also aim to disentangle the mechanistic interplay between αS accumulation, sirtuin dysregulation, and impaired insulin signaling in PD, to identify which phytomolecules are most effective in counteracting mitochondrial stress, mitophagy defects, and neuronal apoptosis. Importantly, several phytochemicals demonstrate metabolic benefits by enhancing GLP-1/GIP signaling through DPP-IV inhibition, providing a biologically plausible route for synergistic neuroprotection in PD (Malar et al., 2020; Singh et al., 2020; Zhu et al., 2017, Alli-Oluwafuyi et al., 2019). Given that GLP-1 receptor agonists and dual GLP-1/GIP mimetics are already in clinical development and have shown beneficial effects on dopaminergic neuron survival, motor function, and pathology in PD patients and models (Naik et al., 2025; Xue et al., 2017; Olszowy-Tomczyk and Wianowska, 2024; Wang et al., 2011; Zhang et al., 2018; Kumar et al., 2006; Guo et al., 2016), they represent a promising disease-modifying therapeutic class.

Therefore, future clinical strategies should investigate whether phytomolecules, either independently or in combination with GLP-1/incretin mimetics, can provide enhanced neuroprotection and improve both motor and non-motor outcomes in PD. Additionally, comprehensive evaluation of long-term safety, pharmacokinetics, bioavailability, and drug-interaction profiles remains essential before such combinational or adjunctive therapies can be translated into clinical PD management (Balakrishnan et al., 2021; Shahpiri et al., 2016; Niu et al., 2025).

## Glossary

5-HT2A: 5-Hydroxytryptamine (Serotonin) Receptor 2A
Aβ: Beta-amyloid
ACO1: Aconitase 1
AICAR: 5-Aminoimidazole-4-carboxamide Ribonucleotide
Akt: Protein Kinase B
ALS: Amyotrophic Lateral Sclerosis
AMPK: AMP-Activated Protein Kinase
ASC: Apoptosis-Associated Speck-like Protein Containing a CARD
ATP: Adenosine Triphosphate
Bax: Bcl-2-Associated X Protein
BBB: Blood–Brain Barrier
cAMP: Cyclic Adenosine Monophosphate
CD11b: Cluster of Differentiation Molecule 11b
COX-2: Cyclooxygenase-2
COMT: Catechol-O-Methyltransferase
CREB: cAMP Response Element-Binding Protein
CSP1: Carbamoyl Phosphate Synthase 1
Drp1: Dynamin-Related Protein 1
DPP-IV: Dipeptidyl Peptidase-4
EGCG: Epigallocatechin Gallate
ER: Endoplasmic Reticulum
ETC: Electron Transport Chain
FoxO1/FoxO3a: Forkhead Box Protein O1/O3a
GIP: Glucose-Dependent Insulinotropic Polypeptide
GLP-1: Glucagon-Like Peptide-1
GSDMD: Gasdermin-D
GSH: Glutathione
GSK3β: Glycogen Synthase Kinase 3 Beta
**H**_**2**_ **O**_**2**_: Hydrogen peroxide
H3: Histone H3
HDAC: Histone Deacetylase
HO-1: Heme Oxygenase-1
IAPP: Islet Amyloid Polypeptide
IL-1β: Interleukin-1 Beta
IL-18: Interleukin-18
iNOS: Inducible Nitric Oxide Synthase
IRP1: Iron Regulatory Protein 1
IRS: Insulin Receptor Substrate
LC3: Microtubule-Associated Proteins 1A/1B Light Chain 3
MAO-B: Monoamine Oxidase B
MIC60: Mitochondrial Contact Site and Cristae Organizing System Subunit 60
Miro1: Mitochondrial Rho GTPase 1
MPTP: 1-Methyl-4-phenyl-1,2,3,6-tetrahydropyridine
MSA: Multiple System Atrophy
mtDNA: Mitochondrial DNA
NAD+: Nicotinamide Adenine Dinucleotide
NADH: Nicotinamide Adenine Dinucleotide (Reduced Form)
NF-κB: Nuclear Factor Kappa-Light-Chain-Enhancer of Activated B cells
NLRP3: NOD-Like Receptor Pyrin Domain-Containing 3
NO: Nitric Oxide
NSF: N-Ethylmaleimide-Sensitive Factor
OGG1: 8-Oxoguanine DNA Glycosylase-1
PD: Parkinson’s Disease
PGC-1α: Peroxisome Proliferator-Activated Receptor-Gamma Coactivator-1 Alpha
PI3K: Phosphatidylinositol 3-Kinase
PINK1: PTEN-Induced Putative Kinase 1
PIP3: Phosphatidylinositol (3,4,5)-Trisphosphate
PRMT6: Protein Arginine Methyltransferase 6
ROS: Reactive Oxygen Species
SGLT1/2: Sodium–Glucose Cotransporter-1/2
Sirt/Sirts: Sirtuins (NAD + -dependent deacetylases)
SNARE: Soluble NSF Attachment Protein Receptor
SNCA: Synuclein Alpha (gene encoding α-Synuclein)
SNpc: Substantia Nigra pars compacta
TCA Cycle: Tricarboxylic Acid Cycle
TLR4/TLRs: Toll-Like Receptors
TOM20: Translocase of Outer Mitochondrial Membrane 20
TPPP/p25α: Tubulin Polymerisation-Promoting Protein
T2DM: Type 2 Diabetes Mellitus
UPR: Unfolded Protein Response
αS: α-Synuclein
